# Safety of Cerebral Intra-Arterial Chemotherapy for the Treatment of Malignant Brain Tumours

**DOI:** 10.3390/jcm14020524

**Published:** 2025-01-15

**Authors:** Gérald Gahide, Jean-François Vendrell, Karine Massicotte-Tisluck, Samuel Caux, Samuel Deschamps, Maxime Noël-Lamy, François Belzile, Laurent-Olivier Roy, David Fortin

**Affiliations:** 1 Department of Medical Imaging, Division of Interventional Radiology, Centre Intégré Universitaire de Santé et de Services Sociaux de l’Estrie—Centre Hospitalier Universitaire de Sherbrooke, 3001 12e Avenue Nord, Sherbrooke, QC J1H 5H3, Canada; karine.massicotte-tisluck@usherbrooke.ca (K.M.-T.); samuel.caux2@usherbrooke.ca (S.C.); samuel.deschamps@usherbrooke.ca (S.D.); maxime.noel-lamy@usherbrooke.ca (M.N.-L.); francois.belzile@usherbrooke.ca (F.B.); 2 Centre de Recherche du Centre Hospitalier Universitaire de Sherbrooke, Universitaire de Sherbrooke, 12e Avenue Nord, Porte 6, Sherbrooke, QC J1H 5N4, Canada; david.fortin@usherbrooke.ca; 3 Institut de Cancérologie de Montpellier, Clinique de Val d’Aurelle, 34090 Montpellier, Cedex 5, France; jfvendrell@yahoo.fr; 4 The Health Campus, Université de Sherbrooke, 3001 12e Avenue Nord, Immeuble X1, Sherbrooke, QC J1H 5N4, Canada; 5 Department of Surgery, Division of Neurosurgery and Neuro-Oncology, Centre Intégré Universitaire de Santé et de Services Sociaux de l’Estrie—Centre Hospitalier Universitaire de Sherbrooke, 3001 12e Avenue Nord, Sherbrooke, QC J1H 5H3, Canada; laurent-olivier.roy@usherbrooke.ca

**Keywords:** cerebral intra-arterial chemotherapy, complications, metastasis, primary brain tumours, primary central nervous system lymphoma

## Abstract

**Background**: Cerebral intra-arterial chemotherapy (CIAC) has been demonstrated to achieve tumoricidal concentrations in cerebral tumour cells that are otherwise unachievable due to the presence of the blood–brain barrier. In this study, we sought to analyze the safety of CIAC in a cohort of patients treated at the Centre intégré universitaire de santé et de services sociaux de l’Estrie—Centre hospitalier universitaire de Sherbrooke (CIUSSS-CHUS). **Methods**: Treatments consisted of monthly CIAC. A neurological examination and neuroimaging study (MRI) were performed before every treatment. The files of patients enrolled in our CIAC programme were reviewed. Adverse events were analyzed and categorized. **Results**: Overall, 2991 CIAC procedures were performed in 642 patients. Pathologies were as follows: malignant gliomas (68.7%), cerebral metastasis (17.6%), and cerebral lymphomas (13.7%). Perfusion vessels were as follows: 80% internal carotid artery and 20% vertebral artery. The chemotherapeutic agents used were carboplatin (86.4%), methotrexate (28.5%), melphalan (28.6%), and liposomal doxorubicin (2.8%). Osmotic blood–brain barrier disruption (BBBD) was induced in 30.5% of treatments. Symptomatic vascular adverse events occurred during 27 procedures (0.9%) in 26 patients (4%). Namely, 23 strokes, one carotid artery occlusion (responsible for one of the strokes), and two intratumoral and one subdural hemorrhage. The absolute risk of stroke was 1.3% and 0.5% for CIAC with or without BBBD, respectively. The use of the vertebral artery significantly increased the risk of stroke. Drug infusion-related seizures occurred in 2.5% of patients; 83.8% were associated with methotrexate and 16.2% with carboplatin. **Conclusions**: CIAC is a safe procedure with a 0.9% overall rate of symptomatic complications (stroke, carotid occlusion, subdural hemorrhage or intratumoral bleeding—n = 27/2991) on a treatment basis, mainly consisting of strokes (85%, n = 23), with a modified NIH Stroke Scale score of 4.1 ± 3.3.

## 1. Introduction

Central nervous system (CNS) malignancies are notoriously difficult to treat. Regardless of whether it is glioma, brain metastases, primary CNS lymphoma, or any other malignancy affecting the CNS, therapeutic approaches are limited by the particularities and characteristics of the blood–brain barrier (BBB). The BBB greatly limits the delivery of chemotherapeutic agents to tumour cells. This limitation restricts the number of agents available for this particular indication. Over the years, a number of alternative delivery strategies have been developed to circumvent this obstacle [1,2].

One of these approaches produces increased drug delivery to the CNS: cerebral intra-arterial infusion of chemotherapy (CIAC). It involves the intra-arterial administration of chemotherapeutic agents into the vascular distribution of the tumour. This paradigm is being explored for the treatment of cancers of many different organs. Indeed, some of these approaches are currently recognized as the gold standards by the medical community for the treatment of neoplasms such as retinoblastoma and liver tumours; in the case of liver tumours, the delivery strategy is called transarterial chemoembolization (TACE) instead of CIAC, but it uses intra-arterial delivery nonetheless [3,4]. The rationale behind this technique is based upon bypassing the first-pass effect, causing an increase in the local plasma peak concentration of the drug to improve the area under the curve (the concentration of the drug according to time) [5,6]. Consequently, this translates into increased local exposure of the target tissue to the therapeutic agent. The drug concentration is therefore increased by 3.5- to 5-fold [5,6,7].

Interestingly, according to preclinical studies, the systemic redistribution of the drug could also be lowered by 20%, potentially reducing systemic toxicity and side effects [1,8].

The CIAC strategy was dismissed many decades ago because of an exceedingly high rate of adverse effects. Indeed, following the initial use of nitrosureas, which elicited up to a 25% rate of complications, the approach was largely abandoned. It has since been realized that the choice of therapeutic agent was the key factor in triggering toxicity, rather than the route of delivery. Because of pharmaceutical advancements, the development of less neurotoxic drugs such as carboplatin and innovations in endovascular techniques allowing minimally invasive approaches, the CIAC paradigm for the treatment of brain tumours has reemerged in the field [8].

In 2001, we initiated a programme of CIAC treatment for CNS neoplasms as part of an integrated preclinical and clinical research effort. Since then, we have been actively involved in the development and refinement of this treatment paradigm [9,10]. In this study, we sought to analyze the safety rather than the efficacy of the CIAC procedure. We report the complication rates for the entire cohort of patients treated at our institution and draw conclusions regarding its overall safety when performed in specialized centres.

## 2. Materials and Methods

### 2.1. Patients and Ethics Approval

The patients reported herein were involved in one of three different studies approved by the Ethics Committee of the CIUSSS de l’Estrie-CHUS (#00-12, 00-13 and 00-51). This study was performed in line with the principles of the Declaration of Helsinki. Written informed consent was obtained from all individual participants included in the study. All the files of the patients enrolled in these studies as part of our brain tumour delivery programme since January 2001 were reviewed. Demographic data including sex, age and brain tumour histopathologic type were prospectively collected and analyzed.

### 2.2. CIAC Treatment and Tumour Response Evaluation

After enrollment and initial evaluation, patients received CIAC every four weeks (one cycle). The procedure was carried out in a standardized way reported elsewhere [11]. Magnetic resonance (MR) scans were performed before every treatment to select the cerebral artery used for delivering the CIAC according to the location of the main tumoral burden. Note that computed tomography (CT) scans were performed only when a complication was suspected. When the tumour encompassed several cerebral arterial territories, the delivery vessels were alternated from treatment to treatment. A 4F or 5F catheter was placed in the proximal segment of the internal carotid artery or in the distal V2 segment of the dominant vertebral artery. Chemotherapy was infused at a constant flow selected for each chemotherapy agent to minimize streaming [11]. According to the treated tumour type and the relevant protocol, one or several of the following chemotherapeutic agents were administered sequentially: carboplatin (400 mg/m^2^), melphalan (10 mg/m^2^), methotrexate (5 mg) or liposomal doxorubicin (40 mg/m^2^). The patients were discharged 24 h after the treatment to allow adequate in-hospital hydration. Neurological and general examinations were performed before each CIAC. A summary neurological evaluation was performed routinely every hour post treatment until discharge. The assessment of overall tumoral response was based on tumour evaluation from MR scans obtained before each treatment and interpreted according to the modified Macdonald or RANO criteria.

If any neurological adverse event was clinically suspected following CIAC, a neuroradiological examination was immediately performed. In the case of stroke, neurological status was graded using the modified NIH Stroke Scale (Lyden). To take into account treatment-related anomalies, only new anomalies or increases in the severity of previously present anomalies were considered for the grading.

### 2.3. Blood–Brain Barrier Disruption (BBBD)

In patients with primary central nervous system lymphoma (PCNSL) and young patients (<40 years old) with primary brain tumours, BBBD procedures were considered. BBBD was performed prior to delivering the chemotherapy. The procedure, which has been previously reported, was performed under general anesthesia [11]. A 5F catheter was used. Mannitol [25%] was infused over a 30 s period in the selected artery to open the BBB. An adequate flow rate was chosen to entirely fill the whole vascular tree with mannitol while keeping reflux to a minimum (between 3 and 6 cc/s).

### 2.4. Clinical and Imaging Data

Since 2001, all clinical and imaging data have been prospectively collected from study entry and from every visit during the study in the institutional digital database system. A systematic search of the following keywords was performed through the numeric files of the patients: hemorrhage, bleeding, hematoma, dissection, stenosis, occlusion, ischemia, lacuna, stroke, cerebrovascular accident, seizure, crisis, ictus, convulsion, and epilepsy. Once a relevant keyword was encountered in a report, an initial screening was conducted to rule out false-positive findings (such as for keywords named in the report but declared as being absent). Files with true-positive keywords were then comprehensively reviewed. Corresponding MR or CT scans and digital subtraction angiography (DSA) images were extracted and systematically reviewed. The lesions were analyzed to determine their nature (imaging behaviour, size, location, and temporal evolution) and potential relationship with CIAC treatment. When the relationship between the lesion and CIAC was confirmed, the clinical reports were reviewed to establish whether there were any associated clinical findings.

### 2.5. Statistical Analysis

Continuous variables are expressed as the means ± standard deviation (SD). Dichotomous and categorical variables are expressed as counts and percentages. Continuous variables were compared using Mann–Whitney or Student’s *t*-tests. Categorical data were compared using the chi-squared or Fisher’s exact tests. The level of statistical significance was set at *p* < 0.05.

## 3. Results

### 3.1. Patient Cohorts

**Figure 1:**
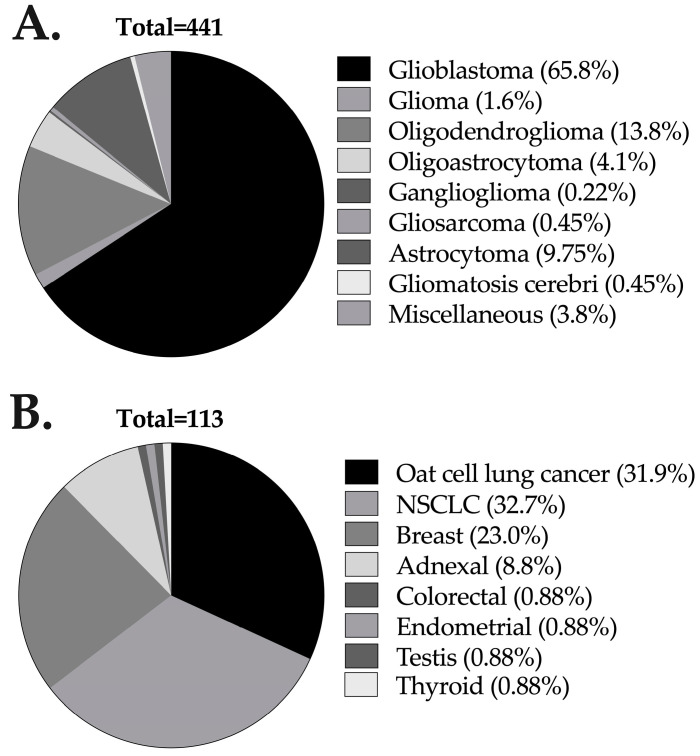
Characteristics and histological subtypes of the cerebral tumours of the primary brain tumours (**A**) and metastases (**B**). Eighty-eight patients (out of a total of six hundred and forty-two) had lymphomas, all of which were central nervous system lymphomas and are thus not displayed in this figure.

A total of 2991 CIAC procedures were performed in 642 patients at our institution, of whom 56.7% were male (n = 364). The mean age of the patients was 50.3 ± 14 years (Table 1). Histopathological diagnoses consisted of primary brain tumours for 68.7% (n = 441), metastases for 17.6% (n = 113) and cerebral lymphomas for 13.7% (n = 88) (Table 1 and Figure 1). The median number of CIAC cycles per patient was 3 cycles (range: 1 to 38). BBBD was performed in 30.5% (n = 911) of the procedures, i.e., for 29.9% (n = 192) of the patients. Most of the CIAC infusions were performed in the internal carotid artery (ICA—n = 2426, 77.9%), whereas the vertebral artery (VA) was the target vessel in 22% (n = 686) of the procedures. A two-vessel approach was used in 4% (n = 121) of cases. The chemotherapeutic agents administered were carboplatin in 86.4% (n = 2585), methotrexate in 28.5% (n = 852), melphalan in 28.6% (n = 854) and liposomal doxorubicin in 2.8% (n = 85) of the CIAC patients.

### 3.2. Report Screening

**Figure 2:**
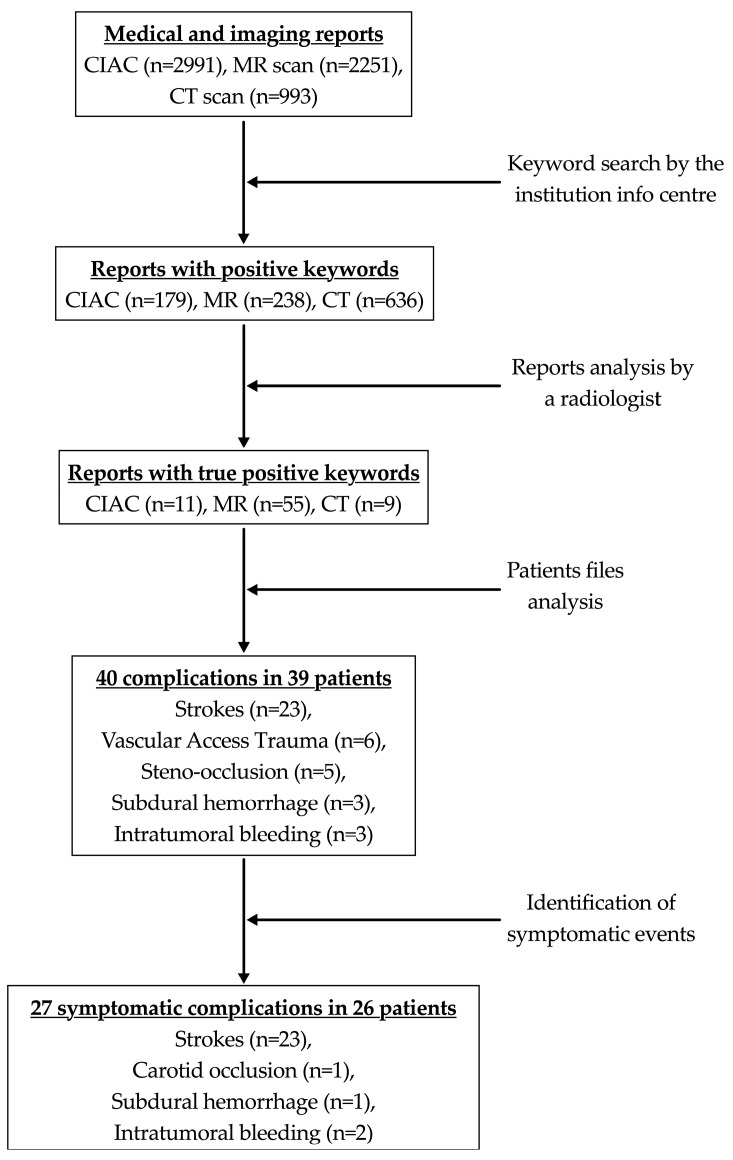
Flow chart illustrating the revision of patients’ medical and imaging reports. CIAC, cerebral intra-arterial chemotherapy; MR, magnetic resonance; CT, computed tomography.

**Figure 3:**
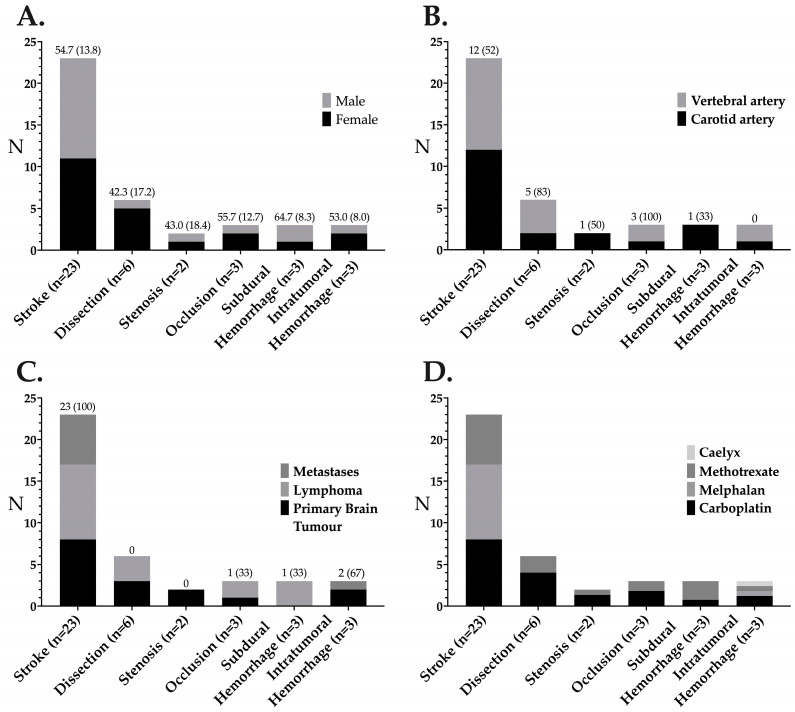
Detailed analysis of the complications encountered in the study. Distribution of complications according to sex with the median age (SD) above the bars (**A**), according to the blood vessel with the number of BBBDs (%) above the bars (**B**), according to brain tumour type with the number of symptomatic events (%) above the bars (**C**) or according to the chemotherapeutic agent used (**D**).

A total of 2991 DSA scans, 2251 MR scans and 993 CT scan reports were screened. The queried keywords were found in 179 DSA scans, 238 MR scans and 636 CT scan reports (Figure 2). After ruling out false-positive reports and carefully analyzing the corresponding images, 40 treatment-related vascular complications were confirmed in 39 patients (1 had a stroke related to carotid artery occlusion), of which 27 were symptomatic (Figure 3).

### 3.3. Adverse Events

The most frequently encountered adverse event was stroke (n = 23). The mean modified NIH Stroke Scale score was 4.1 ± 3.3 (Table 2). The overall risk of stroke was 0.77% per CIAC cycle or 3.6% per patient. For CIAC without and with BBBD, the respective risks of strokes were 0.5% (n = 11/2080) and 1.3% (n = 12/911) per CIAC cycle or 2.4% and 6.3% per patient (*p* = 0.02). There were six asymptomatic vascular traumas, corresponding to six arterial dissections (two carotid and four vertebral arteries). Two asymptomatic significant carotid artery stenoses and three arterial occlusions (one symptomatic carotid artery and two asymptomatic vertebral arteries) occurred. Three subdural hemorrhages occurred, measuring 14 ± 9 mm in thickness, of which one was symptomatic and necessitated surgical evacuation. Three intratumoral bleeding events occurred, of which two were symptomatic (one patient presented with seizures and headaches, and the other presented with seizures and transient hemiparesis). No intraparenchymal or subarachnoid hemorrhage was encountered. Overall, there were statistically more complications when the CIAC was combined with a BBBD (*p* = 0.001), including significantly more strokes (*p* = 0.04), arterial dissections (*p* = 0.01) and occlusions (*p* = 0.03). Vertebral artery perfusion was also associated with an increased incidence of strokes compared to ICA (*p* = 0.001).

### 3.4. Seizures

Seizures occurred during 2.5% (n = 74) of the CIAC cycles in 6.1% (n = 39) of the patients (Table 3). These events were generalized in 12.2% (n = 9) and focal in 87.8% (n = 65) of cases. There were statistically more seizures when using methotrexate (*p* < 0.0001) and in lymphoma patients (*p* < 0.0001). Most seizures were controlled simply by a short pause in the perfusion of the chemotherapy agent.

## 4. Discussion

With an overall symptomatic complication rate of 0.9% on a treatment basis, representing 4% on a patient basis, our study confirms the relatively safe profile of CIAC for the treatment of brain tumours. This complication rate is consistent with those reported for diagnostic cerebral angiography, ranging from 0.3 to 1.3%, and with permanent neurologic complications, ranging from 0 to 0.5% [12,13,14]. Doolittle et al. reported on their experience with the BBBD consortium, a multisite consortium performing CIAC with and without BBBD for malignant brain tumours [15]. These authors concluded that with standardized protocols, CIAC was safe across multiple centres, with a low incidence of catheter-related complications. In their series of 221 patients, treated between 1994 and 1997, they observed a subintimal tear rate of 5%, whereas the rate of strokes was 1.4%.

Strokes were by far the main issue following CIAC, representing 85% of symptomatic vascular complications with a risk of 0.5% per procedure. Importantly, the risk significantly increased to 1.3% when the procedure was combined with BBBD. It is very unlikely that those stroke events were related to direct drug toxicity since most strokes corresponded to very focal lesions on MR scans (Table 3), instead of more evenly distributed signal anomalies in the perfused vascular area, as would be expected with a toxic phenomenon. Since an arteriogram was systematically performed prior to drug delivery, and no arterial defect was visible on any of the 23 arteriograms of the patients who had a stroke, the hypothesis of arterial trauma or plaque mobilization during catheter placement is also very unlikely. These strokes most likely occurred during drug and/or mannitol infusions. Mannitol may crystallize and precipitate when cooled at room temperature. Although every effort is made to avoid this possibility, iatrogenic delay in the perfusion of mannitol could have occurred in some unusual situations. Likewise, some chemotherapeutic drugs may precipitate if not used in a timely manner, which may result in the formation of macroaggregates that could embolize distally. Indeed, melphalan may precipitate when stored at 5 degrees or below. At our institution, it is kept at room temperature and always delivered within 50 min after its preparation. However, despite careful preparation, unforeseen events prior to or during the procedure could have delayed drug administration and been responsible for some of the strokes encountered in our series. Another hypothesis to consider is that thromboembolic events could very likely be responsible for several of the strokes. Indeed, the focal aspect of the lesions on MR scans supports this hypothesis. Moreover, these events were more frequently associated with the use of smaller vertebral vessels (regardless of patient sex) or with BBBD (which requires the administration of several intra-arterial compounds). Though the vessels are carefully examined before every treatment to ensure they are large enough to accommodate a catheter without compromising the blood flow, individual susceptibility to catheters could lead to thrombus formation. Anticoagulation could be considered in patients for whom a long procedural time is expected, such as those with BBBD.

Several important points were raised by this study. First, there were significantly more complications when using the vertebral arteries than when using the carotid arteries, with a relative risk ratio of 3.2 for strokes (11/686 versus 12/2426) and 7.1 for dissections (4/686 versus 2/2426). This is very likely related to the smaller size of those vessels, increasing the risk of arterial wall lesions and atherosclerotic plaque embolization. Interestingly, the proportion of women who suffered strokes or dissection was significantly different (48% and 83%, respectively). This may indicate two different mechanisms for both complications. The significantly higher number of women in the dissection group could be related to a direct trauma of smaller vessels. The catheters used in our study were vertebral-shaped 4F or 5F catheters (for BBBD) for the vast majority of the procedures. This angled catheter could be slightly displaced distally during high-flow volume delivery, coming into contact with the vessel wall and increasing the traumatic risk. This hypothesis is reinforced by the fact that most dissections (83%) occurred during BBBD interventions that involved an infusion of mannitol at a high flow rate (3 to 6 cc/s). This phenomenon could be minimized by using straight catheters or microcatheters for drug delivery into vertebral arteries. On the other hand, the more evenly distributed stroke events between males and females could be related to a thromboembolic phenomenon.

The significantly increased risk of complications when disrupting the blood–brain barrier indicates that patients who would best benefit from this strategy should be properly identified. Currently, at our institution, BBBD is mainly used for patients with PCNSL, allowing a mean survival time of 46.5 months with an actuarial progression-free survival of 83%, 59%, 56%, 30% and 9% after 1, 2, 3, 5 and 10 years [11]. The main advantage over systemic treatment is the absence of neurocognitive deterioration since there is no additional radiotherapy in our protocol. The main interest in cerebral intra-arterial chemotherapy for the treatment of PCNSL could reside in its high capability to induce complete remission, reported to range from 57.8% to 79% [11,16]. BBBD is also considered for well-responding patients bearing malignant glial tumours at the conclusion of their treatment sessions to consolidate the response already achieved [17]. The occurrence of vascular complications was not dependent on sex, age, the number of CIAC cycles or tumour types/subtypes. In contrast, seizures were encountered significantly more often in lymphomas or when using methotrexate. However, the occurrence of a seizure event during methotrexate CIAC administration did not increase seizure incidence between cycles. It was hence a treatment-exclusive complication. Nevertheless, the frequency of seizure events remained fairly low (2.5%). As such, we believe that the CIAC procedure is safe for every patient that meets the prerequisite clinical parameters and for whom it is advisable.

Those rare but very significant complications have to be balanced with the very dire prognosis of cerebral tumours. Following the Stupp protocol, the current reference treatment for malignant glial tumours, the expected median overall survival for patients presenting with glioblastomas ranges from 12 to 16 months [18,19]. Interestingly, the use of CIAC for relapsing glioblastomas significantly prolonged the median survival time from diagnosis and study entry to 23 and 11 months, respectively [10]. Unfortunately, a phase III trial to demonstrate the advantage of CIAC over systemic treatment is still lacking.

### Future Directions for Delivery to the CNS

The normal BBB prevents the passage of ionized water-soluble compounds with a molecular weight greater than 180 Da [20]. Although the integrity of the barrier is often compromised within and around a brain tumour, this alteration in permeability is variable and heterogeneous. Thus, the BBB nonetheless greatly impedes drug penetration [21]. This leads to an uneven drug distribution, with preferential accumulation in the central necrotic areas of the tumour [5].

Other factors also contribute to CNS delivery impediments. The presence of numerous ATP-binding cassette (ABC) efflux transporters, such as P-glycoprotein (P-gp) and multidrug resistance (MDR) transporters, at the luminal surface of the brain capillaries as well as at the surface of tumour cells further contributes to lowering the actual chemotherapy concentration and exposition time of tumour cells [22].

Conceptually, these physiological factors could all be grouped under the broad concept of the blood–brain barrier and the neurovascular unit. Although this concept is increasingly acknowledged, practical approaches to addressing this limitation are still at the research stage and remain underdeveloped, insufficiently discussed and underused in the field of clinical neuro-oncology [8].

Intra-arterial chemotherapy infusion circumvents the first-pass effect, which substantially enhances the plasma peak concentration and the area under the curve. This translates into a significant increase in the intratumoral chemotherapy concentration. This strategy thus improves the delivery of some chemotherapeutic agents that would otherwise not reach therapeutic levels.

Comparing intra-arterial (IA) and intravenous (IV) deliveries, a 20-fold increase in tumour cell nuclear concentration was observed with carboplatin, reaching 79-fold for liposomal oxaliplatin and 153-fold for liposomal cisplatin. These increases in delivery were even more dramatic when BBBD was added to the IA infusion. Specifically, for carboplatin, the IA + BBBD combination further increased delivery by an 18-fold factor compared to IA alone and a 320-fold factor compared to IV infusion [9].

One concern about using such an approach to maximize CNS delivery is the potential risk of neurotoxicity. It should be noted that the neurotoxicity of CIAC is not primarily related to the technique but to the chemotherapeutic agents used. Numerous reports have detailed the safety profile of several chemotherapeutic agents, such as carboplatin, methotrexate, melphalan and bevacizumab [10,23,24,25,26]. Prior to the use of a new agent for CIAC, a preclinical testing phase should be carried out to exclude potential toxic agents. As an example, preclinical studies showed that Taxol, cisplatin and oxaliplatin were too toxic in CIAC settings, thereby precluding the use of these agents [8].

Moreover, the BBB as an obstacle in the treatment of brain tumours can also be bypassed via the use of nanocarriers [27]. These small transporters (1–100 nm) are fashioned using biocompatible/biodegradable molecules (such as lipids, polymers or hyaluronic acid) and can passively or actively penetrate the BBB to target brain tumours [28]. This compelling strategy also provides benefits with regard to systemic toxicity [29]. Consequently, numerous groups have engineered such nanocarriers to carry radiosensitizers or radioenhancers in order to enhance the efficacy of standard radiotherapy or stereotactic radiosurgery [30]. Likewise, several preclinical and clinical studies investigate the safety and efficacy of nanotransporters loaded with chemotherapeutic agents, some of which, like temozolomide and carboplatin, also have radiosensitizing properties [9,31,32].

## 5. Conclusions

Intra-arterial cerebral chemotherapy is a relatively safe procedure when performed by experienced practitioners in a standardized setting and as part of a research continuum. Carboplatin, methotrexate, melphalan, liposomal doxorubicin and etoposide phosphate are all suitable drug candidates for use in this setting. This could help improve patient survival, as formerly demonstrated with relapsing glioblastomas and PCNSL. This technique should be considered a therapeutic option for malignant brain tumours and be properly evaluated with randomized multicentric prospective trials.

## 6. Study Limitations

This was a retrospective study with potential biases related to the methodology.

## Figures and Tables

**Table 1:** Demographic characteristics of the participants in the study.

Patients (n = 642)	
Age, mean (SD)	50.3 (14)
Male, n (%)	364 (56.7)
Female, n (%)	278 (43.3)
Primary brain Tumour, n (%)	441 (68.7)
Lymphoma, n (%)	88 (13.7)
Metastasis, n (%)	113 (17.6)

**Table 2:** Description of the NIH Stroke Scale and lesion size for the 23 strokes encountered in the study.

NIH Stroke Scale	Number of Patients, n (%)	Lesion Size (mm)	Number of Patients, n (%)
1	5 (12.5)	0–5	1 (4.3)
2	5 (12.5)	5–10	5 (21.7)
3	1 (2.5)	10–15	4 (17.4)
4	4 (10)	15–20	1 (4.3)
5	2 (5)	20–30	3 (13)
6	1 (2.5)	30–40	6 (26.1)
7	2 (5)	40–50	2 (8.7)
8	2 (5)	50–60	1 (4.3)
15	1 (2.5)	>60	0 (0)

**Table 3:** Description of the 74 seizures encountered in 39 patients in the study.

Seizures, n (%) ^1^		Drug, n (%) ^2^		Pathology, n (%) ^3^	
Generalized	9 (12.2)	Methotrexate	62 (83.8)	Primary	12 (30.8)
Focal	65 (87.8)	Carboplatin	12 (16.2)	Lymphoma	23 (59)
				Metastasis	4 (10.2)
Total	74		74		39

^1^ Respective numbers of generalized and focal seizures. ^2^ Drug associated with these seizures. ^3^ Tumour pathologies associated with these seizures.

## Data Availability

The datasets used and/or analyzed in the current study are available from the corresponding author upon reasonable request.
